# The Role of T Cell Costimulation via DNAM-1 in Kidney Transplantation

**DOI:** 10.1371/journal.pone.0147951

**Published:** 2016-02-03

**Authors:** Anna K. Kraus, Jin Chen, Ilka Edenhofer, Inga Ravens, Ariana Gaspert, Pietro E. Cippà, Steffen Mueller, Rudolf P. Wuthrich, Stephan Segerer, Guenter Bernhardt, Thomas Fehr

**Affiliations:** 1 Institute of Physiology, University of Zurich, Zurich, Switzerland; 2 Division of Nephrology, University Hospital Zurich, Zurich, Switzerland; 3 Institute of Immunology, Hannover Medical School, Hannover, Germany; 4 Institute of Surgical Pathology, University Hospital Zurich, Zurich, Switzerland; 5 Department of Molecular Genetics and Microbiology, Stony Brook University, New York, New York, United States of America; University of Lisbon, PORTUGAL

## Abstract

DNAX accessory protein-1 (DNAM-1, CD226) is a co-stimulatory and adhesion molecule expressed mainly by natural killer cells and T cells. DNAM-1 and its two ligands CD112 and CD155 are important in graft-versus-host disease, but their role in solid organ transplantation is largely unknown. We investigated the relevance of this pathway in a mouse kidney transplantation model. CD112 and CD155 are constitutively expressed on renal tubular cells and strongly upregulated in acutely rejected renal allografts. *In vitro* DNAM-1 blockade during allogeneic priming reduced the allospecific T cell response but not the allospecific cytotoxicity against renal tubular epithelial cells. Accordingly, absence of DNAM-1 in recipient mice or absence of CD112 or CD155 in the kidney allograft did not significantly influence renal function and severity of rejection after transplantation, but led to a higher incidence of infarcts in CD112 and CD155 deficient kidney allografts. Thus, DNAM-1 blockade is not effective in preventing transplant rejection. Despite of being highly expressed, CD112 and CD155 do not appear to play a major immunogenic role in kidney transplantation. Considering the high incidence of renal infarcts in CD112 and CD155 deficient grafts, blocking these molecules might be detrimental.

## Introduction

Antigen recognition via the T cell receptor is not sufficient for a complete T cell activation. A collection of costimulatory and coinhibitory signals modulates the complex interaction between T cells and antigen presenting cells (APCs) in the process of T cell priming and between T cells and target cells in the effector phase of the immune response [1, 2]. Because of the fundamental role of T cell costimulation in the activation of donor reactive T cells after transplantation, costimulation blockade has become a promising target for the development of more specific and less toxic strategies to prevent rejection and induce tolerance [3]. Latest developments in clinical studies focused on the classical costimulatory molecules B7 and CD40, but additional costimulatory receptors attracted attention as potential targets.

**Fig 1:**
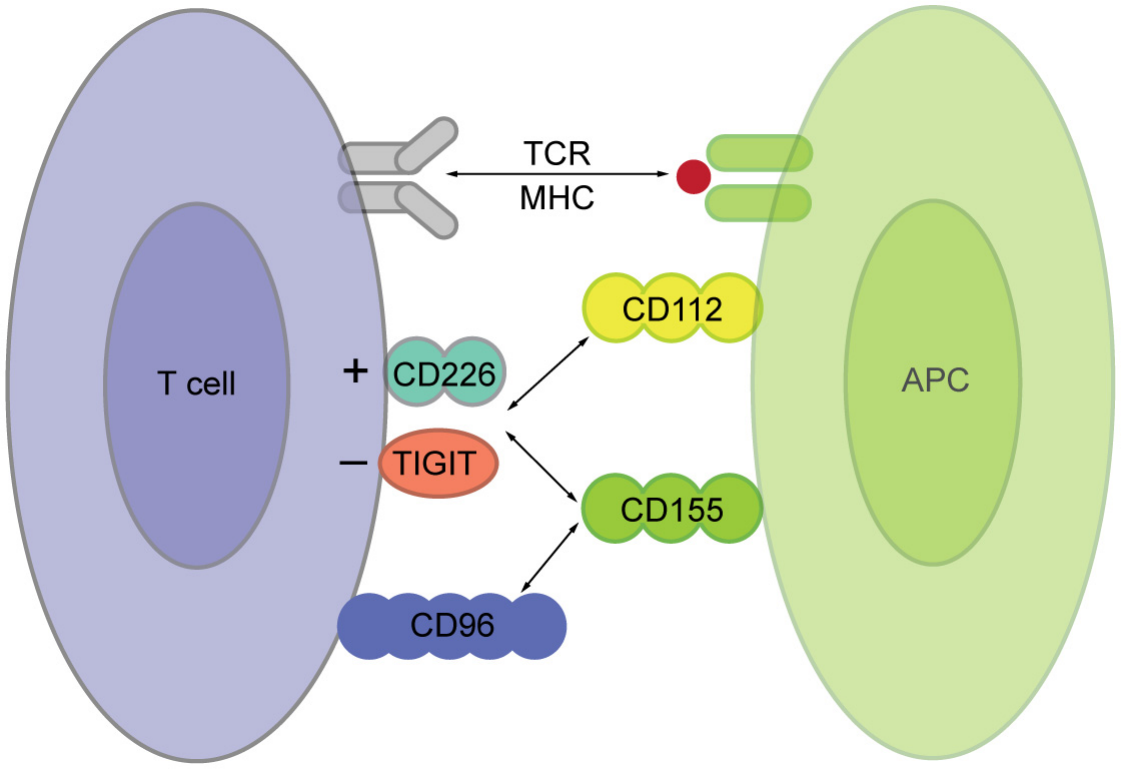
DNAM-1 and its two ligands. Schematic illustration of the DNAM-1 pathway in the interaction between a T cell and an antigen presenting cell (APC). The T cell recognizes its cognate antigen in the context of MHC with its T cell receptor (TCR). For further activation it needs costimulatory signals, which can be delivered via DNAM-1 (CD226) binding to its two ligands CD155 and CD112 expressed on the APC. CD155 and CD112 also bind TIGIT, a co-inhibitory receptor of the Ig-family. CD155 has an additional receptor called CD96.

DNAX accessory molecule-1 (DNAM-1, CD226) has first been described in the 1990s as an adhesion molecule of the immunoglobulin (Ig)-family [4], expressed mainly on T cells and natural killer cells [5]. DNAM-1 participates in proliferation and differentiation of CD4 T cells [6, 7], and particularly in priming and cytotoxic activity of CD8 T cells against non-professional APCs, such as tumor cells [8, 9]. Moreover, DNAM-1 ligation is important for function and differentiation of natural killer cells [10, 11] and mediates platelet adhesion to endothelial cells in particular conditions [12]. DNAM-1 has two known ligands CD155 (Necl-5, PVR) and CD112 (nectin-2) (Fig 1). Both molecules belong to the nectin-family of cell adhesion molecules and are expressed on a variety of epithelial, endothelial, and antigen presenting cells [9, 13–15]. CD155 has a higher affinity to DNAM-1 than CD112 [5, 16]. Both DNAM-1 ligands also bind to T cell Ig and ITIM domain (TIGIT, Vstm3) (Fig 1) [17]. TIGIT belongs to the Ig-family and acts as a coinhibitory receptor on natural killer and T cells [17–19]. An additional player in this complex network is CD96 (TACTILE), which is expressed on T cells and natural killer cells and binds to CD155 and also acts as a co-inhibitory molecule [10, 20].

The absence of DNAM-1 on donor cells reduced graft versus host disease after bone marrow transplantation [21, 22], but the relevance of this pathway in solid organ transplantation is largely unknown. In this study we investigated the role of DNAM-1 and both of its ligands for allospecific T cell priming and cytotoxicity against renal tubular epithelial cells (rTECs) *in vitro* and in a mouse kidney allotransplantation model.

## Materials and Methods

### Mice

C57BL/6 (B6, H-2^b^), CBA (H-2^k^), BALB/c (H-2^d^), DBA/2 (H-2^d^), B6.C-H2-K^bm1^/By (bm1, H-2^bm1^), CD155 KO (H-2^d^) [23], CD112 KO (H-2^b^) [24] and DNAM-1 KO (H-2^d^) mice were bred and housed in specific pathogen-free conditions at the University of Zurich and at Hannover Medical School. Bm1 mice express the same H-2 haplotype as B6 (H-2^b^) except for 7 nucleotide differences in the gene for H-2K^b^ resulting in amino acid substitutions at codons 152 (glutamate to alanine), 155 (arginine to tyrosine) and 156 (leucine to tyrosine) [25]. All animal experiments (including the number of mice, the methods of surgery and anesthesia and the post operative care schedule) were performed according to protocols approved by the legal authorities (Veterinary Office of the Canton of Zurich). The mice were euthanized by CO_2_ inhalation. Since the transgenic mice were available on different genetic backgrounds, different strain combinations were used. In each experiment the appropriate control group in the same strain combination was included.

### Culture of renal tubular epithelial cells (rTECs)

Preparation and primary culture of rTECs was performed as previously described [26]. Cells were cultured on collagen coated dishes in K1 media. In all cytotoxicity experiments primary rTECs were stimulated for 48 hours with murine interferon-β (IFN-β) and IFN-γ at 100 U/ml each (Antigenix America Inc., Huntignton Station, NY, USA), prior to use.

### T cell proliferation and cell-mediated lympholysis (CML) assay

T cell proliferation and CML assays were performed using either whole spleen or isolated CD4 and CD8 positive T cells as responders. Splenocytes were sorted by magnetic cell separation (MACS) according to the protocols of Miltenyi Biotec (Bergisch Gladbach, Germany). Purity of sorted cells was confirmed by FACS analysis. Purity of T cell subpopulations was usually >90%. T cells were stimulated with irradiated (30 Gy) splenocytes from allogeneic and syngeneic mice.

T cell proliferation was measured by incorporation of tritium-labeled thymidine (Perkin Elmer, Waltham, USA) on day 4 of culture. CML assays were performed on day 5 of culture: ^51^Chromium (Cr)-labeled, IFN-stimulated allogeneic rTECs were added to the serially diluted culture for 4 hours (killing phase), and allospecific cytotoxicity was assessed by measurement of ^51^Cr release in the supernatant. Allospecific lysis was calculated as: % specific lysis = (experimental release—spontaneous release) / (total release—spontaneous release) * 100.

In some assays blocking antibodies against mouse DNAM-1 (3B3, [5]) or CD112 (6B3, [27]) or the respective isotype controls were added to the culture.

### Fluorescence activated cell sorting (FACS) and cytokine quantification

FACS was performed with a BD-FACSCanto II (Becton Dickinson, Allschwil, Switzerland). Anti-mouse CD3-FITC, CD4-PE, CD8-APC, anti-rat-IgG-FITC, and propidium iodide (PI) were purchased from eBioscience (Frankfurt, Germany). Rat anti-mouse CD155 was purchased from Biolegend (Fell, Germany) and rat anti-mouse CD112 from Santa Cruz Biotechnology (Heidelberg, Germany). IFN-γ in cell culture supernatants was quantified using a Ready-Set-Go!^®^ ELISA kit purchased from eBioscience (Frankfurt, Germany) according to the manufacturer’s manual.

### mRNA isolation and quantitative PCR

mRNA was isolated from kidney grafts or naïve kidneys stored in RNase-inhibitor using the RNeasy Mini Kit (Qiagen, Hombrechtikon, Switzerland) according to manufacturer’s instructions. mRNA (1 μg) was transcribed to cDNA using the Omniscript reverse transcription Kit (Qiagen, Hombrechtikon, Switzerland) according to manufacturer’s instructions. Pre-developed TaqMan reagents were used for quantitative PCR (Applied Biosystems, Carlsbad, CA, USA) detecting murine CD155 and CD112, and the reference 18s rRNA. The expression of candidate genes was normalized to the reference and fold changes were calculated in relation to the matching controls using the 2^-ddCt method.

### Skin and kidney grafting

For skin transplantations, recipient mice were anesthetized with ketamine/xylazine, pain treated with carprofen (NSAID) and shaved. At day 0 full thickness tail skin (about 0.5–1.0 cm^2^) from donor mice was transplanted to the dorsal flank area of recipient mice. After surgery the general conditions of the mice and the skin graft were monitored daily. Graft rejection was defined as graft necrosis >90% of the graft. Kidney transplantation was performed as described previously [28].

### Histology and immunohistochemistry

Histologic examination of all kidney grafts was performed by an experienced renal pathologist blinded to the experimental procedures. Tissues were immersion-fixed in 4% phosphate-buffered formalin and embedded in paraffin. The thickness of sections was 4 μm. The slides were routinely stained with hematoxylin and eosin (H&E), periodic acid-Schiff-reaction (PAS), and Elastica-van Gieson stain. To quantify the amount of necrotic area in renal allografts H & E stained slides were scanned using the Nanozoomer scanner (Hammatsu, Hammatsu City, Japan) at a resolution of 0.23 μm. The necrotic areas were then quantified using NDPView software (Hammatsu, Hammatsu City, Japan). For detection of apoptotic cells by immunohistochemistry the monoclonal antibody F7-26 (Chemicon, International, Inc. Temecula, CA, USA) was used as previously described [29]. Dense, apoptotic nuclei positive for single stranded DNA were quantified in mouse renal allografts in 15 high power fields (original magnification ×250).

For detection of CD155 by immunohistochemistry a rabbit polyclonal antibody against murine CD155 purchased from Biorbyt (Cambridge, UK). Immunohistochemistry was performed as previously described [30].

### Statistical analysis

All statistical comparisons were performed with GraphPad Prism 4. Groups were compared using Student’s t test or Mann-Whitney test. P < 0.05 was considered as significant.

## Results

### CD112 and CD155 are expressed in renal allografts

**Fig 2:**
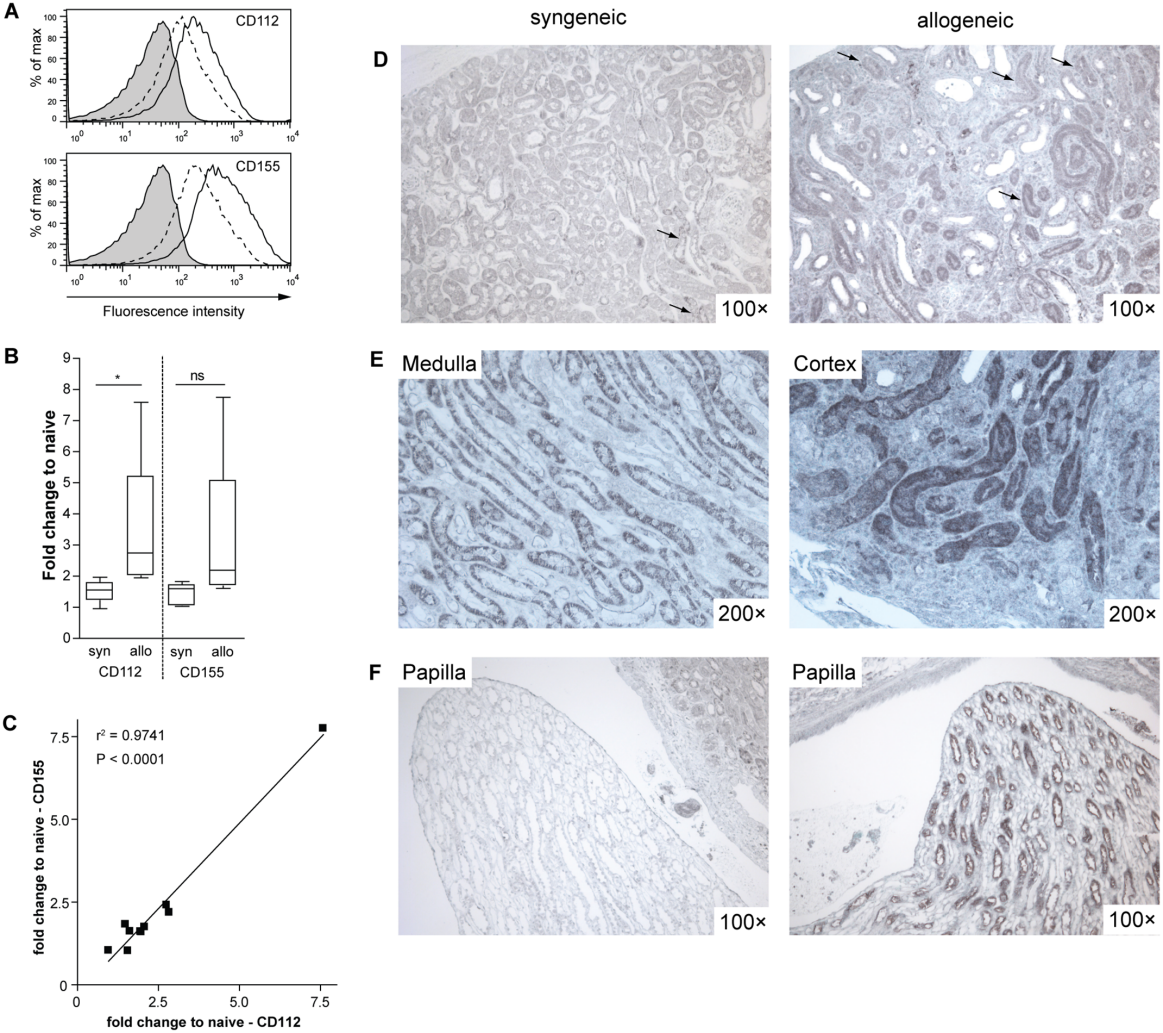
Renal tubular epithelial cells express CD112 and CD155. (A) Primary B6-derived rTECs were left untreated or stimulated with IFN-β and -γ (100 U/ml each) for 48 hours. Surface expression of CD155 and CD112 was analyzed by FACS. Shaded: isotype control, dotted line: unstimulated, solid line: stimulated. A representative result of 4 independent experiments is shown. (B-F) Renal allografts from B6 to fully MHC-mismatched CBA recipients were performed. B6 to B6 syngeneic renal grafts were performed as control. In each group we included at least 5 mice. (B) Real time-PCR for CD155 and CD112 was performed on naïve kidneys and renal syn- and allografts. The fold upregulation of CD112 and CD155 compared to naïve renal tissue is depicted. Groups were compared using the Mann-Whitney-test: * P = 0.016, ns = not significant. (C) The expression of CD155 and CD112 in this group of grafts highly correlated (P<0.001). (D-F) Immunohistochemical staining for CD155 was performed. (D) An overview shows the tubular expression of CD155 in both syn- and allografts (arrows, scale bar 200 μm). (E) The expression of CD155 is located mainly in the medulla in syngrafts and is increased in the cortical area in allografts. (F) The papillae of syngrafts show no staining for CD155, whereas in allografts tubuli in the papillae exhibit strong CD155 staining (scale bar 200 μm).

To assess expression of the two DNAM-1-ligands CD155 and CD112 in renal tissue we first analyzed primary renal tubular epithelial cells (rTECS) isolated from B6 mice and cultured *in vitro*. FACS analysis revealed a constitutive expression of both molecules on primary rTECs and their up-regulation in response to pro-inflammatory cytokines such as IFN-β and IFN-γ (Fig 2A). For a more detailed analysis of the CD112 and CD155 expression after transplantation B6 derived kidney grafts were transplanted into B6 recipients (syngeneic) or into fully MHC mismatched CBA recipients (allogeneic). The kidney grafts were harvested one week after transplantation. Quantitative RT-PCR of whole renal tissue indicated a modest up-regulation of mRNA coding for both CD112 and CD155 in syngeneic grafts compared to naïve kidneys (CD112: 1.52 fold increase, n = 5, p = 0.03; CD155: 1.42 fold increase, n = 5, p = 0.06). The expression of CD112 and CD155 was markedly increased in allografts (CD112: 3.4 fold increase, n = 5, p = 0.08; CD155: 3.1 fold increase, n = 5, p = 0.14; see Fig 2B for differences between syn and allo). Interestingly, we observed a close correlation of CD155 and CD112 expression suggesting a similar regulation for both genes that lie directly adjacent to each other in the genome (Fig 2C). The exact localization of CD155 expression in renal grafts was investigated by immunohistochemistry. CD155 was expressed on tubular epithelial cells in both syngeneic and allogeneic grafts (Fig 2D). The expression of CD155 on tubular cells in syngeneic grafts was concentrated in the medullary region (Fig 2E). In contrast, in allografts CD155 expression was also detected in the cortical region (Fig 2E). Finally, collecting ducts in the papillae of syngeneic grafts did not express CD155 at all, whereas in allografts papillary tubules presented intense staining (Fig 2F). We did not detect an intense endothelial staining in any part of the kidney. Thus, CD112 and CD155 are constitutively expressed in rTECS and their expression increases after transplantation. The functional role of DNAM-1 and its ligands in kidney transplantation was further investigated *in vitro*.

### DNAM-1 blockade inhibits T cell priming independently of CD155/CD112 *in vitro*

**Fig 3:**
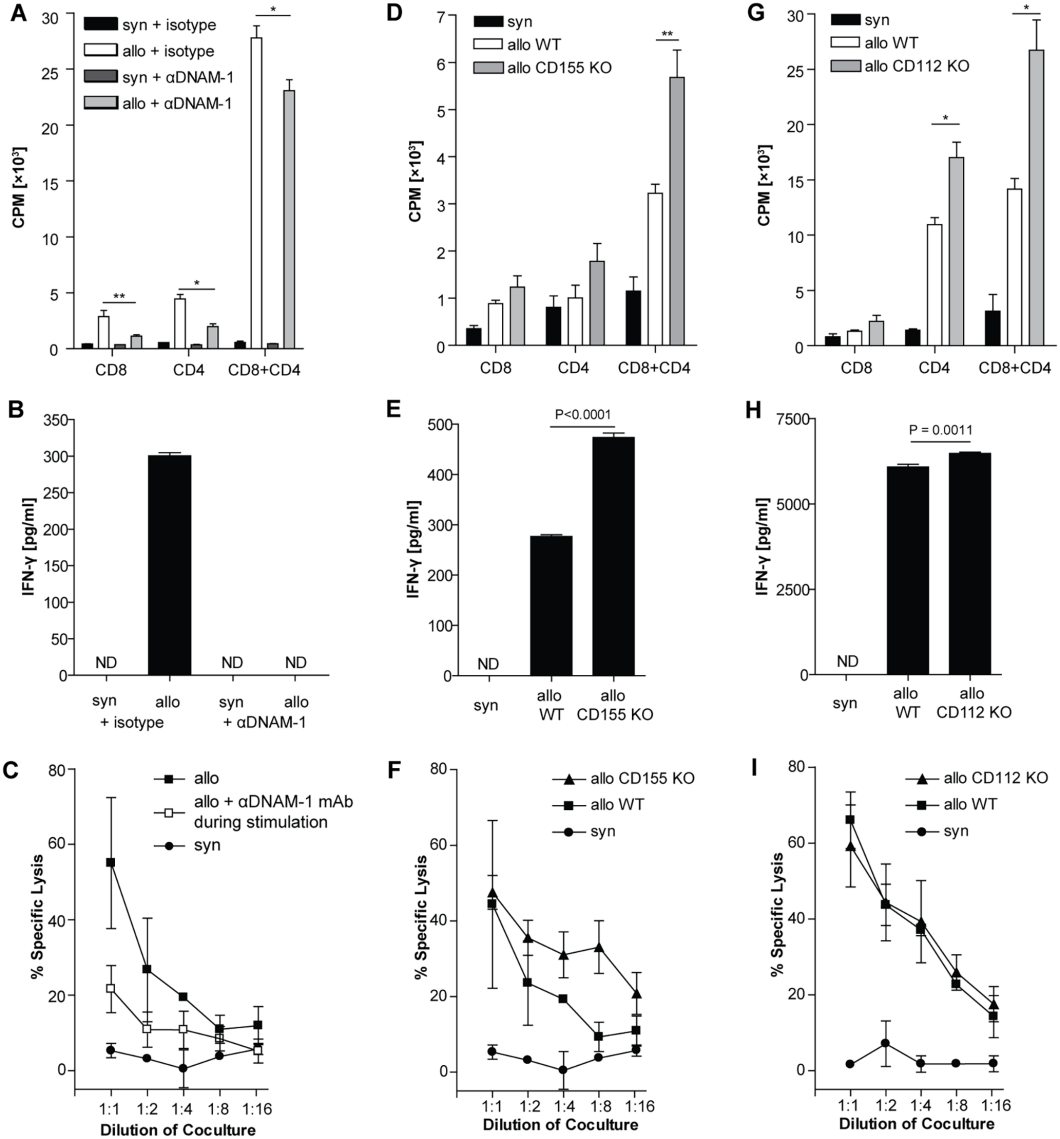
Paradoxical effect of DNAM-1 blockade and CD155/CD112 deficiency. (A-C) Isolated CBA T cells were stimulated with irradiated B6 splenocytes. CD8 or CD4 T cells were either cultured alone or in combination (ratio 1:2). An anti-DNAM-1 antibody (3B3) was added to the culture at 25 μg/ml. As control an unspecific isotype control antibody was used at the same concentration. (A) Proliferation was measured on day 4 of culture. * P = 0.035, ** P<0.01. (B) IFN-γ was measured in the supernatant from the same CD8+CD4 cultures. (C) Cytotoxicity of CBA splenocytes stimulated in the presence or absence of 3B3 (25 μg/ml) against IFN-stimulated B6 WT rTECs was measured on day 5. (D-F) Isolated B6 T cells were stimulated with irradiated BALB/c WT or CD155 KO splenocytes. CD8 or CD4 T cells were either cultured alone or in combination (ratio 1:2). (D) Proliferation was measured on day 4 of culture. ** P<0.01. (E) IFN-γ was measured in the supernatant from the same CD8+CD4 cultures. (F) Cytotoxicity of the CD8+CD4 culture against IFN-stimulated WT BALB/c rTECs was measured on day 5. (G-I) Isolated CBA T cells were stimulated with irradiated B6 WT or CD112 KO splenocytes. CD8 or CD4 T cells were either cultured alone or in combination (ratio 1:2). (G) Proliferation was measured on day 4 of culture. * P = 0.02. (H) IFN-γ was measured in the supernatant from the same CD8+CD4 cocultures. (I) Cytotoxicity of CBA splenocytes stimulated with irradiated WT B6 or CD112 KO splenocytes against IFN-stimulated WT B6 rTECs was measured on day 5. Data points represent mean values of triplicates. All experiments were performed at least 3 times. Representative figures are displayed.

To analyze the role of the DNAM-1 pathway for T cell priming after transplantation, isolated naive T cells were stimulated with allogeneic splenocytes in the presence or absence of an antibody blocking DNAM-1 (3B3). Allospecific T cell proliferation was modestly but significantly reduced under DNAM-1 blockade when using CD8 or CD4 T cells alone or in combination as responder cells (Fig 3A). In the same experimental setting, IFN-γ production was suppressed, when DNAM-1 was blocked (Fig 3B). Furthermore, allospecific cytotoxicity against rTECs was markedly reduced, when T cells were primed in the presence of the DNAM-1 blocking antibody (Fig 3C).

To systematically elucidate the role of the DNAM-1-ligands CD112 and CD155 on T cell priming, wild type T cells were stimulated by splenocytes isolated from CD112 KO or CD155 KO mice. In the absence of either one of the two DNAM-1-ligands on the stimulator cells, allospecific T cell proliferation was not reduced, but rather enhanced compared to MHC-matched controls (Fig 3D and 3G). In parallel, IFN-γ production was significantly increased, when stimulators did not express CD155 or CD112 (Fig 3E and 3H). Finally, cytotoxicity of T cells stimulated with either CD155 or CD112 KO splenocytes against primary wild type rTECs did not significantly differ from those stimulated with WT spleocytes (Fig 3F and 3I).

Thus, DNAM-1 plays a significant costimulatory role during allospecific T cell priming, and this process is not dependent on its classical ligands CD112 and CD155 on APC.

### Allospecific T cell cytotoxicity against rTECs is independent of the DNAM-1 pathway *in vitro*

According to previous reports, the DNAM-1 pathway is important for T cell-mediated killing of non-professional APCs [9]. rTECs are considered important targets of allospecific cytotoxic T cells during renal allograft rejection and act as non-professional APCs under inflammatory conditions [26, 31]. Since they express both CD112 and CD155 after transplantation (Fig 2), we aimed to asses the functional relevance of DNAM-1 signaling in allospecific cytotoxic activity against rTECs in the effector phase of the immune response. To this end, splenocytes were first primed with fully MHC-mismatched splenocytes, after 5 days of culture their cytotoxic activity against WT, CD155 or CD112 KO rTECs was measured *in vitro*.

**Fig 4:**
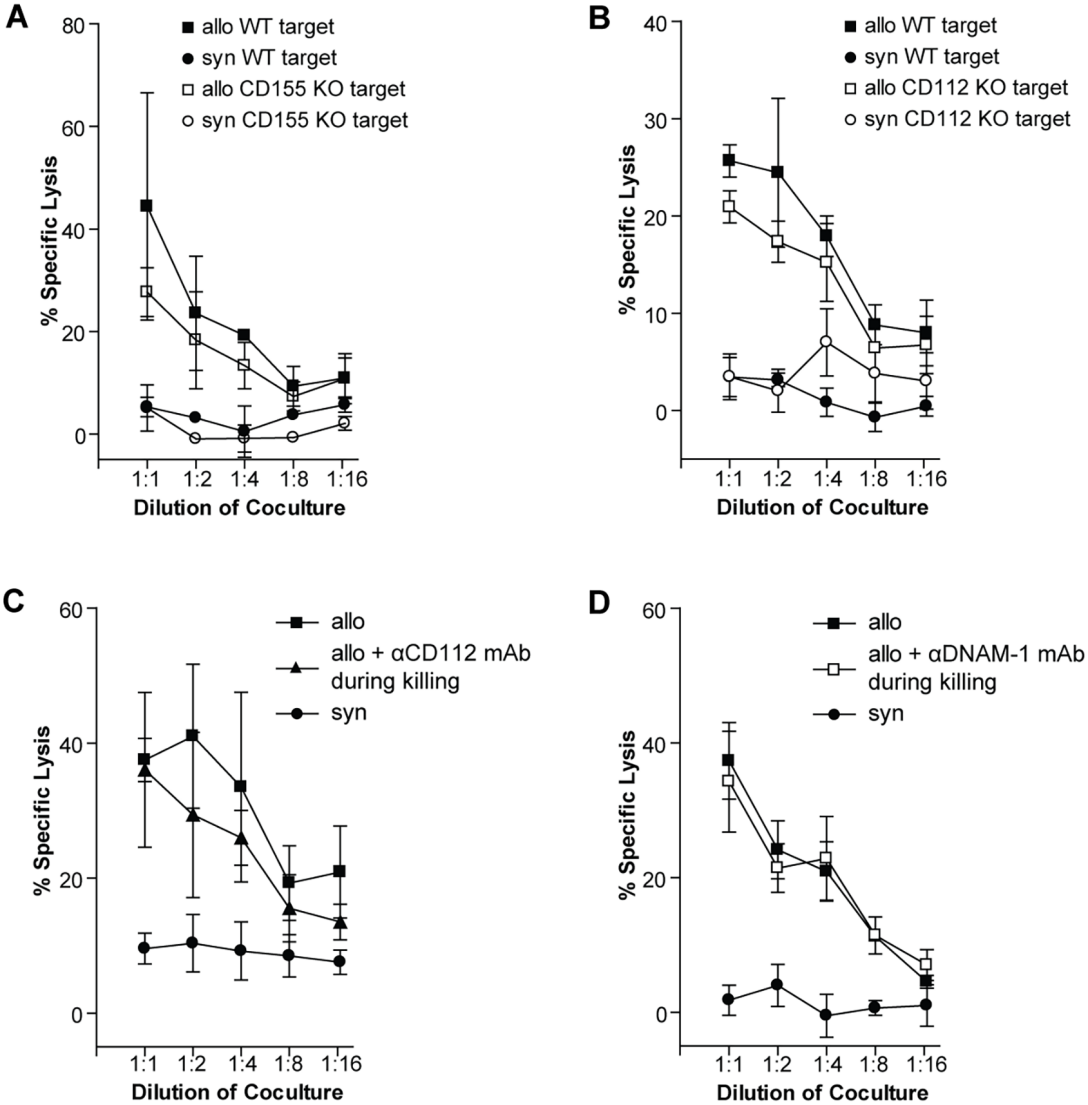
Cytotoxic activity of allospecific T cells against rTECs is not DNAM-1 dependent *in vitro*. (A) CBA splenocytes were stimulated with irradiated BALB/c splenocytes. Cytotoxicity against IFN-stimulated WT BALB/c or CD155 KO rTEC targets was measured on day 5 of coculture. (B) CBA splenocytes were stimulated with irradiated B6 splenocytes. Cytotoxicity against IFN-stimulated WT or CD112 KO targets was measured on day 5 of coculture. (C) CBA splenocytes were stimulated with irradiated BALB/c splenocytes. Cytotoxicity against IFN-stimulated CD155 KO rTEC targets in the presence (25 μg/ml) or absence of a blocking anti-CD112 antibody was measured on day 5 of coculture. (D) CBA splenocytes were stimulated with irradiated B6 splenocytes. Cytotoxicity against IFN-stimulated WT targets was measured on d 5 of coculture in the presence or absence of a blocking anti-DNAM-1 antibody (50 μg/ml). Data points represent mean values of triplicates. All experiments were performed at least 3 times. Representative figures are displayed.

Absence of CD112 or CD155 on the target cells did not alter the killing rate (Fig 4A and 4B). To exclude redundancy between both ligands, we used CD155 KO targets and added a blocking antibody against CD112. Even if none of the two ligands was available for DNAM-1 binding, rTEC killing was not impaired (Fig 4C). Finally, to exclude the possibility of a third unknown ligand binding to DNAM-1 we added the same antibody blocking DNAM-1, which already exerted a significant effect when used during T cell priming (Fig 3). However, this did not reduce allospecific killing of rTECs (Fig 4D), indicating that DNAM-1 is not essential for the cytotoxic effector function of T cells against rTECs *in vitro*.

### Renal transplantation in DNAM-1 deficient mice

**Fig 5:**
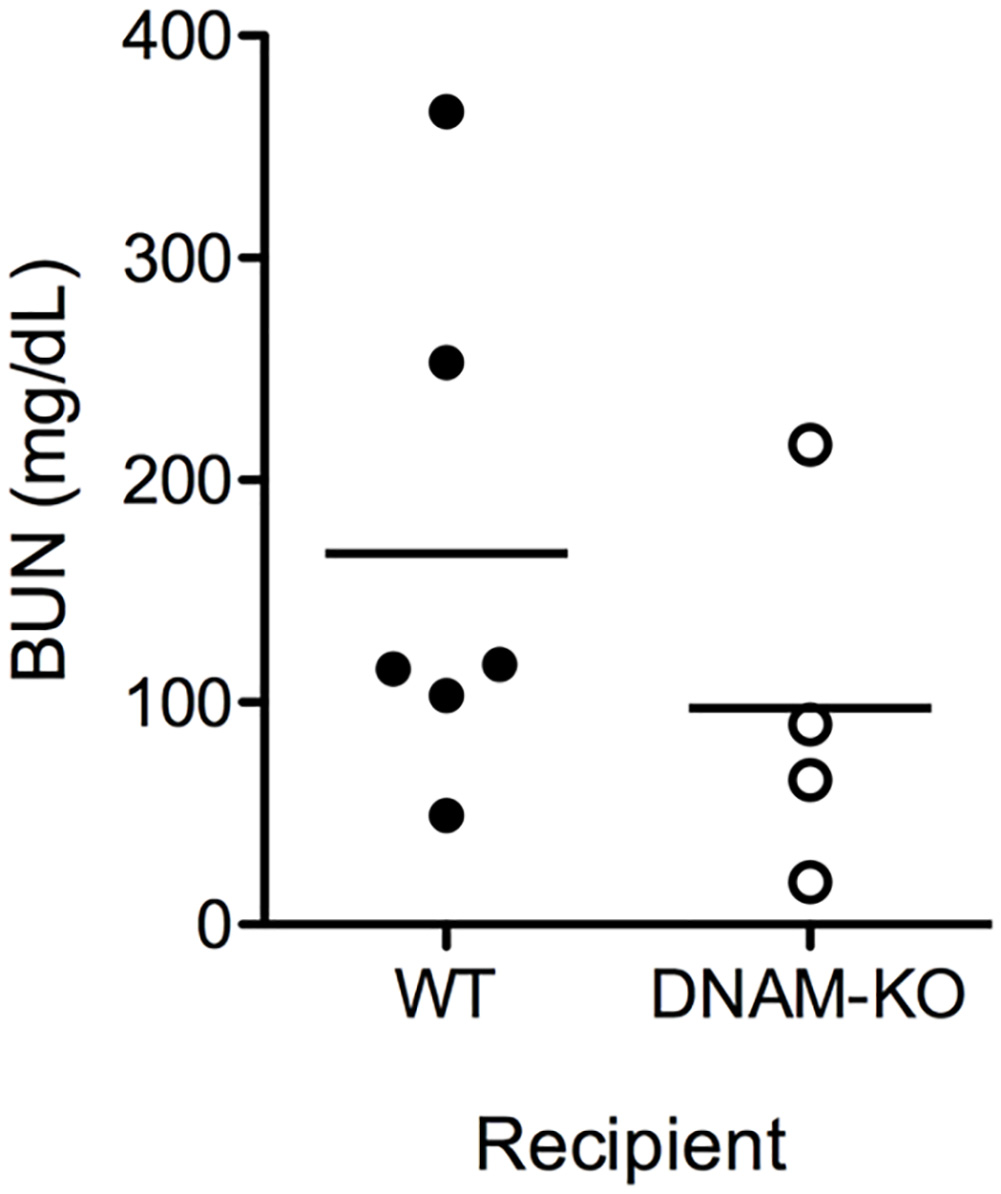
Renal allograft function in DNAM-1 deficient recipients. Renal transplantation was performed in two steps. First, fully MHC-mismatched CBA kidney allografts were transplanted into WT BALB/c or DNAM-1 deficient mice. The second kidney was removed 7 days after surgery and 3 days later renal graft function was estimated by measurement of serum creatinine und urea. Mean BUN WT 167.2 ± 48.4 mg/dl vs. DNAM1-KO 97.5 ± 42.1 mg/dl, P = 0.36. One mouse in the DNAM1-KO group and 2 mice in the WT group had to be excluded because of surgical complications. Thus, in the final analysis 6 mice were included in the WT group and 4 in the DNAM1-KO group.

The specific role of DNAM-1 in kidney transplantation was investigated by transplanting fully MHC-mismatched kidney allografts in DNAM-1 deficient mice. Renal transplantations were performed in a non-life supporting manner, leaving the left kidney untouched and replacing only the right kidney by the allograft. Seven days after non-life supporting kidney transplantation the second kidney was removed, and 3 days later renal graft function was estimated by measurement of serum creatinine und urea. Additionally, the kidney grafts were harvested at day 10 after transplantation for histological analysis. On histological assessment kidney allograft rejection according to Banff classification was not reduced in DNAM-1 KO mice (4 grafts acute vascular cellular rejection Banff IIA, 1 graft with extensive acute tubular necrosis) compared to controls (2 grafts with borderline changes, 1 graft acute tubulointerstitial cellular rejection Banff IA, 2 grafts IIA, 2 grafts with extensive tubular necrosis). Serum creatinine and BUN did not significantly differ between the two groups, but a trend towards a better renal function in DNAM-1 deficient mice was observed after exclusion of kidney grafts with histological evidence for extensive tubular necrosis (mostly related to surgical complications) (mean BUN 97.5 ± 42.1 mg/dl in DNAM-1 KO and 167.2 ± 48.4 mg/dl in WT, p = 0.36, Fig 5). Thus, absence of DNAM-1 did not prevent, but might moderately mitigate rejection after renal transplantation.

### Transplantation of CD112 or CD155 deficient allografts

**Fig 6:**
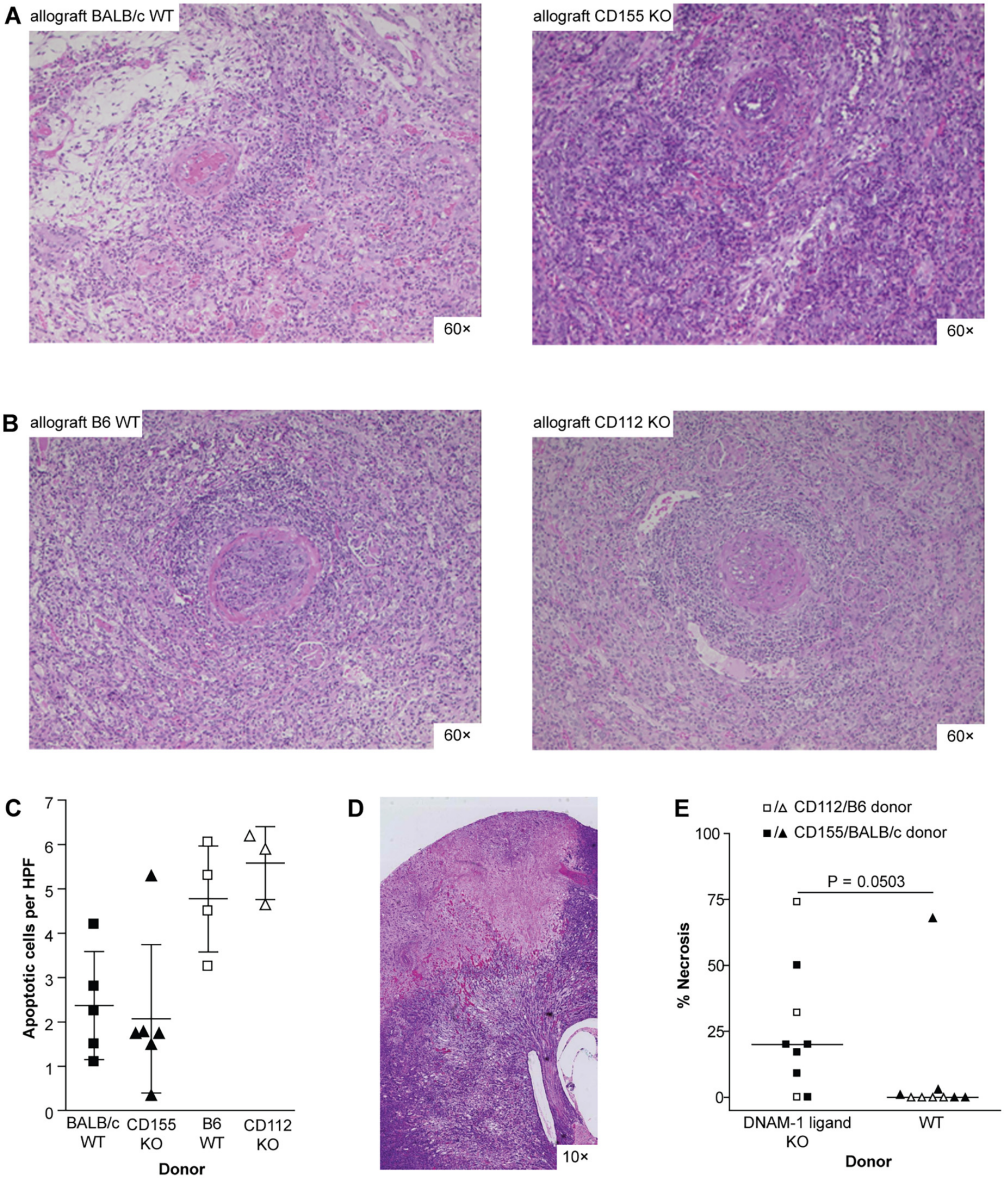
Similar rejection but higher rate of infarcts in renal allografts from CD155 or CD112 KO donors. Renal allografts were performed in a non-life supporting manner. All allografts were harvested on day 21. Strain combinations were fully MHC-mismatched: (A) BALB/c WT (n = 5) or CD155 KO (n = 5) into B6 and (B) B6 WT (n = 5) or CD112 KO (n = 3) into CBA. Representative H & E stainings are shown. All allografts displayed severe interstitial infiltrates as well as tubulitis in more than 50% of the graft. Furthermore, arteritis was detected in all grafts classifying them to Banff grade II or III. The chosen pictures are taken from allografts with the following Banff grades: (A) BALB/c to B6: IIA; CD155 KO to B6: IIB; (B) B6 to CBA: IIB; CD112 to CBA: III (scale bar 200 μm). (C) Apoptotic cells in renal allografts were detected by immunohistochemical staining for ssDNA. (D) Representative picture of a necrotic area in an H & E slide of a CD155 KO renal allograft (scale bar 1200 μm). (E) The area of necrotic tissue in H & E stained slides from renal allografts was detected by scanning them at a resolution of 0.23 μm. Quantification was performed using NDPView software. Groups were compared using the Mann-Whitney-test.

For a comprehensive analysis of this costimulatory pathway in kidney transplantation, we additionally performed transplantation of kidney grafts harvested from CD112 and CD155 deficient mice into MHC mismatched recipients. Renal transplantations were performed in a non-life supporting manner. Allografts harvested on day 21 showed severe rejection with infiltrates in the interstitium and tubulitis. In addition, all allografts demonstrated vascular lesions classifying them to Banff grade II or III rejections. These findings were independent of the expression of CD155 or CD112 in the allograft (Fig 6A and 6B). The amount of apoptotic epithelial cells representing an *in vivo* estimate for the strength of cytotoxic activity was not different, when comparing CD155 or CD112 KO allografts with their respective WT controls (Fig 6C). The absence of a significant functional relevance for CD112 and CD155 in solid allograft was additionally confirmed in a skin transplantation model using a fully MHC-mismatched and a less-stringent single antigen mismatch combination (S1 Fig). Unexpectedly, we observed a high incidence of infarcts and subsequent development of necrosis in allografts lacking CD155 or CD112. Quantification of the necrotic areas in the H&E stained slides revealed a significant difference when comparing WT versus DNAM-1 ligand KO allografts (Fig 6D).

## Discussion

The identification of costimulatory pathways with a functional relevance in allogeneic immune responses is important for the definition of novel pharmacological targets to prevent allograft rejection. We took advantage of our mouse kidney transplantation model to investigate the role of T cell costimulation via DNAM-1 in renal transplantation by using gene-targeted mice. We found that in allogeneic immune responses the DNAM-1 signaling pathway is involved in T cell priming, but not in the effector phase of the immune response. Although CD112 and CD155 were highly expressed in kidney allografts, blocking the DNAM-1 pathway did not prevent rejection of kidney allografts.

Previous studies suggested a role for the DNAM-1/CD155-interaction during T cell priming either to a nominal antigen [9] or allospecifically [21]. Consistent with these results we detected reduced proliferation, cytokine production, and subsequent lower cytotoxic activity against rTECs, when DNAM-1 was blocked during the stimulation of T cells. This was true for both CD4 and CD8 T cells. This effect, however, was independent of the expression of CD112 or CD155 on the stimulator cells. This finding is in line with previous reports demonstrating a role for DNAM-1 signaling in LFA-1 mediated T cell activation [6]. When using CD155 or CD112 deficient cells as stimulators in proliferation and cytotoxicity assays *in vitro*, we observed a paradoxically enhanced proliferation and cytokine production. Furthermore, in a single MHC I antigen mismatched skin transplantation model allograft rejection was accelerated, when CD112 was not expressed on donor skin. These results indicate a regulatory role of CD155 and CD112 during the process of T cell priming. Indeed, blocking CD155 on human vascular endothelial cells augmented the acquisition of effector functions by CD8 T cells [32]. Furthermore, in a GVHD model mortality was increased in CD155 KO recipients [33]. These findings might be explained by the effect of TIGIT. TIGIT is expressed on activated T cells and triggers an inhibitory signal upon binding to CD155 or CD112 [18, 19]. When CD155 or CD112 are missing on the stimulator cells, binding and triggering of TIGIT is also abrogated possibly leading to a stronger T cell response. The exact mechanism for this phenomenon has still to be elucidated. Alternatively, absence of CD155 on stimulator cells also may prevent an inhibitory influence of CD96 that was shown recently to counteract CD226 driven activation.

The lack of a significant role for DNAM-1 pathway in kidney transplantation is quite surprising, since DNAM-1 and its ligands CD155 and CD112 are important in NK and T cell mediated anti-tumor activity [4, 8, 34] and play a role in graft versus host disease [11, 22]. Our interest for this particular pathway in kidney transplantation came out in consideration of the role of DNAM-1/CD155 interaction for the cytotoxic activity of T cells against non-professional APCs [9]. Since rTECs can act as non-professional APCs under inflammatory conditions and expressed both CD112 and CD155, we postulated a critical role for this pathway in kidney allograft rejection. Although a modest (statistically not significant) benefit in kidney transplant function was observed in DNAM-1 deficient recipient (Fig 5), overall, inhibition of the DNAM-1 pathway exerted only a mild effect on allograft rejection. The small number of DNAM-1 KO mice available for kidney transplantation and some experimental variability inevitably related to the complex surgical model preclude a conclusive interpretation of the functional data based on creatinine and BUN levels. Moreover, although the histological examination of the grafts did not show any difference in cellular infiltrates between DNAM-1 and wild type recipients, immunological tests to specifically assess T cell activation and functional activity might be useful for a better characterization of the DNAM-1 pathway *in vivo*. However, we conclude that the the effect of DNAM-1 on T cell priming is of limited functional relevance in the complex immune reaction triggered by kidney transplantation. Since T cell activation after kidney transplantation is primarily mediated by donor-derived professional APCs evading the graft, other costimulatory molecules might be sufficient for a full T cell activation and DNAM-1 might be redundant in this setting. Also the postulated role of CD112 and CD155 for cytotoxic effector function was not confirmed *in vitro* and *in vivo* under the experimental conditions applied in this study.

Furthermore, we unexpectedly observed a higher incidence of ischemic infarcts in CD155 or CD112 deficient allografts compared to their WT controls. Taking into account the pivotal role of nectins in cell-cell adhesion, a less efficient endothelial recovery might predispose those grafts to thrombosis. CD155 is located on the leading edge of moving cells and continues to sustain mobility until contacting another cell [14]. Thus, when ischemia-reperfusion injury causes endothelial damage [35], this might be repaired less efficiently in renal allografts from CD155 and CD112 KO donors. Gaps in the endothelial layer may then expose the subendothelial layer, allow platelet activation and adhesion, and promote clotting of the vessel [36].

Taken together our results suggest a limited role for DNAM-1 in solid allograft rejection. Blocking DNAM-1 is not sufficient to prevent allograft rejection and might be relevant only in combination with additional inhibitors of costimulation. Furthermore, blockade of one of its ligands may even be detrimental after transplantation because of non-immunological functions of CD112 and CD155.

## Supporting Information

S1 Fig Absence of DNAM-1 ligands does not prolong skin allograft survival. (A) Skin grafts from fully MHC-mismatched WT BALB/c (n = 19) or CD155 KO (n = 24) donors were performed on B6 recipients. Median survival time: 9 vs. 10 days (WT vs. CD155 KO). (B) Skin grafts from WT BALB/c (n = 7) or CD155 KO (n = 8) donors were performed on minor antigen mismatched DBA/2 recipients. Median survival time: 13 vs. 15 days (WT vs. CD155 KO). (C) Skin grafts from fully MHC-mismatched WT B6 (n = 8) or CD112 KO (n = 8) donors were performed on CBA recipients. Median survival time: 11 vs. 12 days (WT vs. CD112 KO). (D) Skin grafts from MHC I antigen mismatched B6 (n = 7) or CD112 (n = 8) donors were performed on bm1 recipients. Median survival time: 25.5 vs. 21 days (WT vs. CD112 KO, P = 0.01).
(TIF)
