# Supplementary figures and images for: The Role of T Cell Costimulation via DNAM-1 in Kidney Transplantation
Source: PLoS One. 2016 Feb 3;11(2):e0147951. doi: 10.1371/journal.pone.0147951 (PMC4739582; doi:10.1371/journal.pone.0147951)

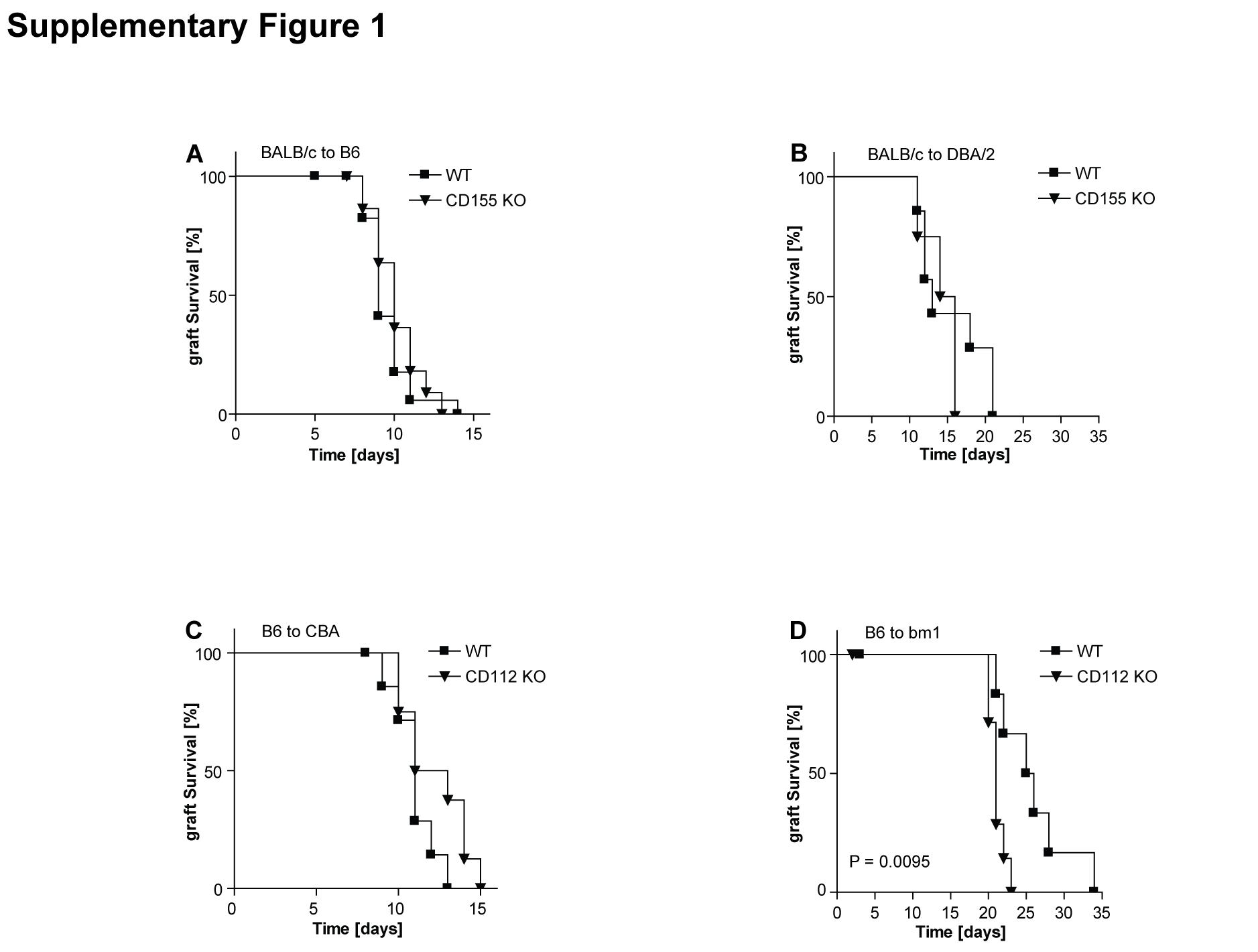

Supplement: S1 Fig — (A) Skin grafts from fully MHC-mismatched WT BALB/c (n = 19) or CD155 KO (n = 24) donors were performed on B6 recipients. Median survival time: 9 vs. 10 days (WT vs. CD155 KO). (B) Skin grafts from WT BALB/c (n = 7) or CD155 KO (n = 8) donors were performed on minor antigen mismatched DBA/2 recipients. Median survival time: 13 vs. 15 days (WT vs. CD155 KO). (C) Skin grafts from fully MHC-mismatched WT B6 (n = 8) or CD112 KO (n = 8) donors were performed on CBA recipients. Median survival time: 11 vs. 12 days (WT vs. CD112 KO). (D) Skin grafts from MHC I antigen mismatched B6 (n = 7) or CD112 (n = 8) donors were performed on bm1 recipients. Median survival time: 25.5 vs. 21 days (WT vs. CD112 KO, P = 0.01). (TIF) [file pone.0147951.s001.tif]
